# Rewiring of the Serotonin System in Major Depression

**DOI:** 10.3389/fpsyt.2021.802581

**Published:** 2021-12-16

**Authors:** Faranak Vahid-Ansari, Paul R. Albert

**Affiliations:** Ottawa Hospital Research Institute (Neuroscience), University of Ottawa Brain and Mind Research Institute, Ottawa, ON, Canada

**Keywords:** serotonin, neuroplasticity, antidepressant (AD), serotonin receptors, axonal guidance and plasticity

## Abstract

Serotonin is a key neurotransmitter that is implicated in a wide variety of behavioral and cognitive phenotypes. Originating in the raphe nuclei, 5-HT neurons project widely to innervate many brain regions implicated in the functions. During the development of the brain, as serotonin axons project and innervate brain regions, there is evidence that 5-HT plays key roles in wiring the developing brain, both by modulating 5-HT innervation and by influencing synaptic organization within corticolimbic structures. These actions are mediated by 14 different 5-HT receptors, with region- and cell-specific patterns of expression. More recently, the role of the 5-HT system in synaptic re-organization during adulthood has been suggested. The 5-HT neurons have the unusual capacity to regrow and reinnervate brain regions following insults such as brain injury, chronic stress, or altered development that result in disconnection of the 5-HT system and often cause depression, anxiety, and cognitive impairment. Chronic treatment with antidepressants that amplify 5-HT action, such as selective serotonin reuptake inhibitors (SSRIs), appears to accelerate the rewiring of the 5-HT system by mechanisms that may be critical to the behavioral and cognitive improvements induced in these models. In this review, we survey the possible 5-HT receptor mechanisms that could mediate 5-HT rewiring and assess the evidence that 5-HT-mediated brain rewiring is impacting recovery from mental illness. By amplifying 5-HT-induced rewiring processes using SSRIs and selective 5-HT agonists, more rapid and effective treatments for injury-induced mental illness or cognitive impairment may be achieved.

## Introduction

Major Depressive Disorder (MDD) is characterized by a persistent low mood as a core symptom. The prevalence of depression is about 1 in 5 of the general population, affecting nearly 300 million people worldwide (1), and its prevalence has increased during the COVID-19 pandemic (2). The most recent global data place MDD as the third greatest source of disability, after low back pain and headache disorders (3). The currently available antidepressant drugs (ADs) have several disadvantages, including delayed efficacy (4–8 weeks) (4), numerous adverse effects that reduce tolerability (0.64- to 0.83-fold) and modest efficacy (1.15- to 1.55-fold) compared to placebo (5, 6) that limit therapeutic effectiveness to ~30% remission (7). Among approved ADs, selective serotonin reuptake inhibitors (SSRIs) are the first-line treatment, and almost all target 5-HT and other monoamine systems (8). However, it is not fully understood why, despite brain levels of serotonin increasing with hours after SSRI administration, behavioral improvement takes weeks to be observed. This delay might reflect neuro-adaptive changes in pre-and post-synaptic cells, including long-term changes in gene expression, protein translation, or ultimately in neuroplasticity (9, 10). As it is diagnosed by a diversity of symptoms in the absence of biomarkers, major depression remains heterogeneous. A better understanding of the mechanism/s underlying the development of depression and its phenotypes will be critical to develop a more efficient, rational clinical approach to targeted treatment (10, 11).

Unraveling the pathophysiology of depression is a complex challenge. Not only are syndromes heterogeneous and the etiology diverse, but important symptoms such as guilt and suicidality cannot be reproduced in animal models. Nevertheless, other symptoms like anxiety, anhedonia, or behavioral despair have been modeled in animals (12–15), and these, together with clinical data, are providing insight into the neurobiology of mood disorders (16, 17) and antidepressant action (18). Recent studies combining behavioral, molecular, and functional imaging in transgenic mice have revealed that alterations in the functional connectivity of specific subpopulations of neurons forming a neural circuit result in depression-like behaviors (19–23). Understanding the underlying causes of these functional changes might offer a crucial new direction for the development of novel treatments for MDD in humans.

## Serotonin Axon Hypothesis in Depression

In pre-clinical and clinical studies, deficits in serotonergic transmission including reductions in serotonin (5-hydroxytryptamine, 5-HT) neurons and their projections and increases in 5-HT autoinhibition have been associated with MDD and also with impaired responses to antidepressants (24–36). 5-HT is a monoamine neurotransmitter found mainly in blood platelets and the central nervous system (CNS) in animals and humans. It is widely implicated in mood, emotion, and happiness (37). The monoamine-serotonin hypothesis for depression was proposed in the 1960s suggesting that brain deficiency of monoamines, including 5-HT, triggers the onset of depression (38–40). It continues to guide research into the causes and treatments for depression, anxiety, and other mental illnesses.

### The 5-HT System

The 5-HT neurons originating in the raphe midbrain innervate several regions of the brain (41–43). In 5-HT neurons, the enzyme tryptophan hydroxylase type 2 (TPH2) converts the amino acid tryptophan to 5-hydroxytryptophan (5-HTP) to catalyze the rate-limiting step in 5-HT biosynthesis (44–46). Subsequently, the L-aromatic amino acid decarboxylase (AADC) enzyme generates 5-HT. Alterations in 5-HT neurotransmission have been implicated in the pathophysiology of depression and its treatment. Based on clinical evidence that depressive symptoms improve following specific blockade of the 5-HT transporter (5-HTT) (47), early research focused on the uptake site at the terminal targets (48). The forebrain projecting raphe nuclei include the dorsal (DRN) and median (MRN) raphe and contain a diversity of 5-HT and non-5-HT neurons, identified using viral-genetic, immunohistochemistry and electrophysiology methods (49–51). For example, a small population of 5-HT immune-positive cells are not co-labeled with 5-HT1A receptors (52, 53) while some non-5-HT cells (such as GABA neurons) are co-labeled (52). Some 5-HT neurons co-express vesicular glutamate transporter-3 conferring glutamate neurotransmission and are implicated in anxiety behavior (54). Importantly, different projections of these neurons to target regions may confer stress susceptibility, depression or anxiety behaviors (36, 55, 56). Thus, the distinct properties of select 5-HT neuronal populations may confer behavioral phenotype and response to stress or injury.

In addition to developmental innervation, a unique capacity of the 5-HT system to regenerate or alter its innervation of brain regions has been observed after neurotoxin, traumatic or ischemic brain damage in rodents (57–64). Changes in 5-HT innervation have also been observed in non-lesion conditions such as repeated stress rodent models of depression (65, 66) and Parkinsonism in rodents (67) and primates (68). In post-mortem brain tissue from human depressed subjects, a reduction in 5-HT innervation has also been seen (32), although the functional role of these changes remains unclear.

The extensive ascending and descending 5-HT network projects throughout the brain and spinal cord making synaptic or non-synaptic contacts that release 5-HT (69–71). Actions of 5-HT are mediated by at least 14 different receptor subtypes (72). It is believed that 5-HT axons prenatally initiate axon outgrowth concomitant with the onset of 5-HT synthesis (73, 74). 5-HT axons form and grow in a targeted manner through guided pathfinding and arborization over several weeks. The 5-HT rich brain areas include cortical and sub-cortical regions. In addition, sensitive HPLC measurements of 5-HT and metabolites have shown that the metabolic activity of 5-HT fibers extending from DRN and MRN is parallels the tissue content of 5-HT (75). Therefore, it is expected that the alterations in 5-HT axons are associated with concomitant changes in 5-HT levels in the same brain region.

### Development of 5-HT Projections

A large body of studies has characterized the molecular determinants involved in the developmental mechanisms targeting raphe 5-HT projections to the forebrain, proposing that alterations in these processes may predispose to mood disorders (74). Many of the transcription factors in the 5-HT gene regulatory network required for differentiation and maintenance of 5-HT neuronal subgroups have been extensively characterized, including Lmx1B, Pet-1/FEV, and others (76, 77). These factors may also be involved in axonal outgrowth as shown for Lmx1B (78); conditional deletion of Lmx1B in 5-HT neurons resulted in the loss of axonal projections to the forebrain and spinal cord in mice. Cytoskeleton-associated proteins growth-associated protein 43 (GAP-43) and a microtubule-associated protein, stable tubule only polypeptide (STOP) are also required for the growth and elongation of the 5-HT axons (76). In normal mice, GAP-43 is prenatally expressed on growing 5-HT axons; but in GAP-43 knockout mice, there is a loss of 5-HT immunoreactive innervation of the cortex and hippocampus (79). In the STOP knockout mice, 5-HT levels, as well as 5-HTT density and terminals, are reduced in projection areas such as hippocampus, but increased in the raphe suggesting impaired trafficking of 5-HT vesicles resulting in deficits in hippocampal neurogenesis, reduced anxiety, increased helplessness, and impaired cognitive function (80, 81). Despite these deficits in 5-HT innervation, no significant difference in the number of dorsal raphe 5-HT neurons was observed in GAP-43 or in STOP knockouts compared to wild-type littermates. These results suggest that GAP-43 and STOP proteins are the key regulators of normal 5-HT outgrowth and innervation in healthy conditions.

Concerning 5-HT axon pathfinding and guidance, Fenstermaker et al. (82) reported that Wnt signaling to planar cell polarity components is required for anterior to posterior axon projection and for proper orientation of 5-HT cell bodies in the raphe nuclei, using mice lacking individual planar cell polarity genes (82). In addition, to guide the 5-HT axons along the midline and form the long-distance connectivity both Robo1/2 and Slit1/2 have key roles in the formation of major forebrain tracts as shown in knockout mouse lines (83, 84).

Genes involved in cell adhesion have also been implicated in 5-HT axonal outgrowth in development, including the Pcdh-α isoforms (85, 86). In particular, loss of the αC2 isoform in serotonergic neurons, but not in 5-HT target brain regions, leads to abnormal projection and tiling of serotonergic axons, associated with increased depression-like behaviors (87). Interestingly, differentiation of induced pluripotent stem cells from SSRI-resistant compared to SSRI-responsive depressed patients to a serotonergic phenotype resulted in deficiencies in Pcdh-α expression and neurite outgrowth *in vitro* (88). More recently, an epigenome-wide association study of 150 monozygotic twins reported 428 differentially methylated genes associated with early-onset major depression, many of which are implicated in neurodevelopmental and cell adhesion genes including the protocadherin-α (Pcdh-α) gene cluster (89). Taken together these studies implicate Pcdh-α genes in 5-HT axonal outgrowth, major depression and response to SSRI antidepressants.

## Visualizing Serotonin Axons

### Early Markers

Early studies of 5-HT projections in the brain relied on a relatively insensitive formaldehyde-induced immunofluorescence method to visualize 5-HT (90). Subsequent studies used labeling with [3H]5-HT or immunostaining for 5-HT to visualize 5-HT axons in brain sections (71, 91). However, more recent studies have used the more sensitive approach of 5-HTT immunostaining to visualize 5-HT projections (92). In addition, antibodies to TPH have been useful, particularly in human post-mortem brain sections (41). These studies have revealed that 5-HT fibers rarely branch and have a high density in many brain regions. A high density of 5-HT projections has been shown in the cerebral cortex and subcortical regions including striatum, hippocampus, entorhinal cortex and the NAc [core and caudal shell (93)]. In a single fiber, there are several specialized boutons or varicosities where 5-HT is concentrated (91). It has been estimated that there are around 6 × 10^6^ varicosities/mm^3^ in the rat cortex. In addition, each cortical neuron may receive around 200 varicosities (94, 95). However, there is evidence of some non-5-HT producing neurons that transiently express 5-HTT during development in the thalamus identified by 5-HT uptake and *in situ* hybridization for 5-HTT RNA (96). Using 5-HTT-cre mice to drive reporter gene expression, labeling was seen in dorsal thalamus, cingulate cortex, hippocampal CA3 neurons, retinal ganglion cells, superior olivary and cochlear nuclei during embryonic development and postnatally in medial prefrontal cortex (97). These studies suggest that neurons that lack TPH can take up 5-HT and in the thalamus can store the 5-HT in vesicles for co-transmission with glutamate. On the other hand, chronic SSRI-induced blockade of 5-HTT leads to uptake and release of 5-HT by the dopamine transporter in DA neurons (98, 99). Similar, l-DOPA treatment leads to DA uptake and release in 5-HT neurons (100), indicating cross-talk between monoamine systems at the level of co-transmission following chronic drug treatment.

### Non-synaptic 5-HT/Volume Transmission

In addition to conventional synapses, serotonin is also released from varicosities into extracellular spaces with no target cell dendrites nearby (70, 101), a process termed volume transmission (102, 103). The non-synaptic release of 5-HT allows a paracrine transmission of serotonin to distal neurons and glia, particularly in the presence of 5-HT reuptake blockers, to activate high-affinity 5-HT receptors. Thus, both synaptic and non-synaptic 5-HT release may be implicated in the actions of raphe activation. As discussed below, activation by 5-HT of multiple 5-HT receptors engage several effector proteins to regulate neurite outgrowth, growth cone motility, synaptogenesis, and shape the dendritic spine and density in a wired brain.

The above examples illustrate that to identify 5-HT neurons and their projections it is important to combine different approaches. Recently, several genetic approaches using transgenic mice 5-HT-specific promoters (including 5-HTT, TPH2, Pet-1) to drive the expression of reporter genes (such as LacZ, YFP) have been used in combination with immunostaining for 5-HT markers (5-HT, 5-HTT, TPH2) to identify 5-HT projections (104, 105). These labeling approaches have been combined with anterograde and retrograde labeling techniques (106) to further define at a macroscopic level the neuroanatomy of the 5-HT system (56, 107–111). For example, at the cellular level, dual retrograde tracing revealed that a small (10–20%) proportion of neurons innervate both nucleus accumbens and medial prefrontal cortex (112). Single-cell biotin labeling has also been used to localize region-specific 5-HT/vGlut3 projections (113). These results indicate that single 5-HT neurons can innervate multiple brain regions.

### Visualizing 5-HT Synapses

For high-resolution visualization of 5-HT synapses, electron microscopic (EM) studies (91) and 3D reconstruction of 5-HTT-positive axons have been used to map the 5-HT boutons located proximal to excitatory or inhibitory synapses in limbic brain regions (114, 115). Post-synaptic components of excitatory or inhibitory synapses form “triads”. To finely dissect how 5-HT exerts its modulatory actions, asymmetrical synapses/excitatory triads were mostly localized in the hippocampus, cortex, mPFC while symmetrical synapse/inhibitory triads were enriched in the dorsal raphe nucleus (DR), ventral tegmental area (VTA), central and basolateral amygdala (CeA, BLA) (116, 117). The combination of both (excitatory-inhibitory) was observed in areas like thalamic regions, bed nucleus of stria terminalis (BNST), and nucleus accumbens (NAc) (91, 114, 115). The preferential proximity of 5-HT boutons to neurochemical excitatory/inhibitory synapses could therefore suggest that serotonergic axons projecting to one area may preferentially target local glutamatergic, interneurons, or both to modulate their activity. For example, the preferential proximity of 5-HT-positive axon terminals to GABA terminals engaged in symmetrical synapses in DR and amygdala sub-regions (CeA, BLA) suggests that 5-HT mainly modulates the activity of interneurons in DR and amygdala. In contrast, 5-HT axon terminals are mainly engaged in asymmetrical synapses in mPFC to modulate the activity of excitatory neurons. Therefore, alterations in 5-HT system activity which preferentially change the activity of different cell types in target brain regions could differentially impact behavioral output. Recently, using a semi-automated approach that combines immunohistochemistry and high-resolution confocal microscopy to label 5-HTT immunoreactive axons has allowed researchers to reconstruct the 5-HT axons in 3D through their distribution within limbic brain regions (114, 115). Using this approach, the changes in 5-HT axon properties have been also determined in a model of post-stroke depression induced by focal ischemia in mice medial prefrontal cortex (mPFC), before and after treatment with chronic fluoxetine (64).

Imaging techniques have shown that the density and other features of 5-HT fibers can be altered during and after development. For example, Azmitia et al. (118) found that the density of serotonergic fibers is unusually high in the cerebral cortex of individuals suffering from autism spectrum disorders (118). In contrast, post-mortem studies in adult subjects showed that depression is associated with reduced 5-HT innervation of the orbitofrontal cortex in addition to the loss of hippocampal volume (32). Liu and Nakamura (65) reviewed the effects of chronic stress on regeneration of noradrenaline (NA) and 5-HT axons following NA or 5-HT neurotoxin in adult rats (65). They reported that, in contrast to NA axons, 5-HT axons are more dynamic in morphological plasticity as they are easily affected by stress and rapidly regenerate after damage. 5-HT axons also exert an inhibitory effect on NA axon regeneration. Furthermore, in a depression model induced by 9-week administration of interferon-α to adult male Sprague-Dawley rats, the density of 5-HT-stained axons decreased specifically in the ventral medial prefrontal cortex and amygdala (119). Thus, using new imaging approaches could promote the early diagnosis and development of more effective treatments for depression based on the morphological plasticity of 5-HT axons.

## Volume Transmission: Beyond Synaptic Communication in the Wired Brain

Recent scientific evidence has focused on the complexities of neurotransmitter (NT) communication in the wired brain. In this regard, the importance and relevance of both fast-targeted synaptic and slow-non synaptic transmission has been recognized.

The concept of non-synaptic communication or volume transmission in the brain was proposed in the 1980s (102, 120), and shown for monoamines including 5-HT (117, 121). In 1994, Bjelke et al. showed indirect evidence that amphetamine-induced dopamine release may diffuse long distances following fiber tracts, possibly to the contralateral hemisphere (122). This is supported by the diffusion of Texas-Red-labeled dextran injection in the striatum, which diffuses along fiber tracts to the contralateral brain hemisphere (123). More recently, based on the half-life of dopamine it has been calculated that it might diffuse up to 7 microns (124). With newer, more sensitive indicators specific for dopamine and other monoamines (125), it may be possible to detect the diffusion of dopamine from non-synaptic release.

Using techniques such as receptor autoradiography, immunohistochemistry, and EM imaging has shown for monoamines a mismatch between the location of NTs relative to synaptic structures (103, 126, 127). For example, Martin et al. (128) showed that 94% of tyrosine hydroxylase-positive boutons in macaque prefrontal cortex Area 10 had no identifiable synaptic association in non-human primates (129). Rice et al. (130) modeled dopamine release to show that the presence of dopamine outside of the synaptic zone in the nigrostriatal pathway could be due to the spillover from the synaptic cleft and release into the surrounding extracellular space. Dopamine concentration remains sufficiently high to activate extra-synaptic dopamine receptors on surrounding cells (130). Mapping studies using diverse techniques also identified varicosities filled with NT granules localized along the axons. This evidence supports the existence of NTs in a high volume in non-terminal axon segments. Rodent studies showed that the main action of modulatory NTs including acetylcholine, norepinephrine, dopamine, and serotonin in the brain is through volume transmission via non-synaptic contacts of varicosities within axons (126).

After the first evidence supporting the concept of volume transmission in dopamine release in the brain by Fuxe and Ungerstedt (131), similar approaches were used for the 5-HT cell bodies located in dorsal raphe upon treatment of rats with 5-HT reuptake blocker clomipramine (132). The release of 5-HT from vesicles in the soma, dendrites, and/or axonal varicosities could also be independent of targeted synapses (133–135). More directly, parachloroamphetamine-induced non-synaptic somatodendritic release of 5-HT has been visualized using 3-photon microscopy of dorsal raphe sections (136). Somatodendritic and axonal release of 5-HT can be triggered by neuron depolarization, the stimulation of L-type calcium channels, activation of glutamatergic receptors, and/or by activation of 5-HT2 receptors (137). Furthermore, somatodendritic 5-HT release can also regulate the rate of discharge of serotonergic neurons and their tonic activity, via somatodendritic 5-HT1A and 5-HT2B autoreceptors (29, 138, 139). Nevertheless, direct evidence of 5-HT volume transmission-induced depression of 5-HT firing has not been reported (140). However, it has been recently shown that somatodendritic release of dopamine acting via D2 receptors autoinhibits the firing of the same neuron (141), suggesting a truly auto-regulatory system.

## Axonal and Neurite Outgrowth: Serotonin Receptors

During development, differentiation of 5-HT neurons (e10.5–13 in rat) and outgrowth 5-HT projections is initiated early in embryonic development of the brain (e12–14 in rat) and continues well into post-natal development (p21 in rat) (73, 76, 142, 143). The availability of serotonin during embryonic to early post-natal developmental stages implicate serotonin signaling in directed axonal and neurite outgrowth during development (144, 145) and also in mediating neuroplasticity responses to external stimuli during and post-development (146). In this light, deletion of TPH2 to block neuronal 5-HT synthesis results in abnormal projections of 5-HT neurons both during development and in adults (147–150). Serotonin can also accumulate in (96, 151) and affect the development of non-serotonergic neurons in cortex and hippocampus (147, 152–155). Vicenzi and Gasperini (156) recently found that exogenous serotonin acts as a guidance cue during axon pathfinding in sensory neurons *in vitro*, capable of concentration-dependent attraction (via 5-HT2A receptor) or repulsion (via 5-HT1B receptor) of growth cone motility (156). However, the role of endogenously released 5-HT gradients in axonal outgrowth *in vivo* remains to be assessed.

The actions of serotonin on target cells, including glutamate and GABA neurons, are mediated by a large family of 5-HT receptors. Currently, genes for 14 receptors, including 13 distinct heptahelical G protein-coupled receptors (GPCRs) and one ligand-gated ion channel, have been identified. Based on their structural and downstream signaling characters, receptors are divided into seven distinct classes including 5-HT1-7 receptors (72, 157). The role of some key receptors in the 5-HT axonal transmission including axonal growth and axonal guidance is summarized below.

### 5-HT1 Receptors

The largest class of 5-HT receptors is the 5-HT1 receptor family characterized by an intronless coding sequence with five subtypes sharing 40–63% sequence homology (72, 157). The 5-HT1A,−1B,−1D,−1E and−1F receptors are localized in a wide variety of brain regions and show distinct pharmacological characteristics. The 5-HT1A receptors are broadly expressed in cortex, limbic areas, raphe nuclei (on 5-HT neurons as autoreceptors), in extrapyramidal areas, such as the substantia nigra, caudate-putamen, and in the cerebellum during embryonic-early postnatal development (158–168). The 5-HT1A receptors have been also found on astrocytes (169, 170) to mediate neuroprotective actions (171).

Using *in vivo* studies, Azmitia et al. (172) showed that 5-HT1A receptors have a key role in 5-HT-induced increases MAP2 and synaptophysin in the hippocampus, hypothalamus, parietal and temporal cortices, and the temporal pole (172). *In vitro* studies showed that 5-HT1A receptor stimulation decreased neurite outgrowth in cortical neurons (173), increased it in hippocampal cultures (174) while had no effect or inhibit outgrowth in serotonergic raphe neurons (175, 176). The 5-HT1A receptor can trigger diverse downstream signaling mechanisms that are region- and cell-specific and may mediate these actions (177, 178). 5-HT1A receptor coupling via Gβγ subunits reduces neuronal activity by opening potassium channels and closing calcium channels. However, the receptor is coupled primarily to Gi3 in 5-HT neurons and Gi2 in hippocampal neurons, which may underlie differential signaling and desensitization in these cells. While in 5-HT neurons, the 5-HT1A receptor appears to inhibit extracellular regulated protein kinase (ERK) ERK1/2 activity (179), it signals to activate it in developing and adult hippocampal neurons and may play roles in synaptogenesis (180). Recent studies implicate 5-HT1A signaling through Gβγ and tyrosine kinase receptors to activate ACII (181), phospholipase C (PLC)/protein kinase C (PKC) (182), calcium-calmodulin-dependent protein kinase II (CAMKII) (183), and phosphatidylinositol 3'-kinase (PI3K)/Akt signaling (184) mediating synaptogenesis, dendrite outgrowth, cell survival. Thus, the 5-HT1A receptor appears to modify its signaling repertoire depending on the cell type (5-HT vs. post-synaptic neurons) and the developmental state of the neuron (178). Previous studies also showed the crucial role of serotonin in modulating the neuronal guidance cues to shape the connectome in the wired brain mediated by the 5-HT1 family (185, 186). For example, 5-HT1B/1D receptor activation induces the growth and guidance of embryonic thalamocortical axons (187). In this process, axon responses to netrin-1 shift from attraction to repulsion during the cortical network shaping.

Cortical plasticity in adulthood can also be modified by 5-HT1 receptor activity. For example, chronic fluoxetine treatment induced a full recovery from monocular deprivation in adult rats by increasing brain-derived neurotrophic factor (BDNF) expression to reduce GABAergic activity in the visual cortex thus enhancing excitatory long-term potentiation (188). These actions of fluoxetine suggest that synaptic, possibly structural re-organization of the cortex can be induced in adulthood. Interestingly, these actions of fluoxetine were blocked by 5-HT1A antagonist WAY-100635, implicating 5-HT1A-induced BDNF expression in adult cortical plasticity (189). It remains unclear whether similar 5-HT1-induced signaling to BDNF in the PFC may mediate synaptic reorganization implicated in the antidepressant actions of SSRIs as seen for rapidly acting antidepressants such as ketamine (190, 191).

### 5-HT2 Receptors

The 5-HT2 receptor subtypes including 5-HT2A-C share about 50% amino acid sequence identity and show similarities concerning molecular structure, pharmacology, and signal transduction pathways (72, 192). 5-HT2A receptor expression is widely observed in cortical areas (neocortex, entorhinal, and piriform cortex), olfactory tubercle, dentate gyrus, and several brainstem nuclei, motor cranial nerve nuclei, and the spinal cord ventral horn (168, 193). *In vitro* studies have shown that stimulation of 5-HT2A receptors inhibits neurite growth in serotonergic neurons (176) while increasing neurite outgrowth in thalamic neurons (194), with no effects on cortical glutamatergic neurites (195). Recent studies by Vicenzi et al., (156) using a growth cone motility assay showed that serotonin is capable of acting as both attractive and repulsive guidance cue on its own axons via activation of 5-HT2A and 5-HT1B receptors, respectively (156). The low concentration of serotonin as 50 μM induces attraction mediated by 5-HT2A while twice this concentration elicits the repulsion through the stimulation of 5-HT1B. In agreement, high-resolution imaging of growth cones indicateds that differential signaling is involved. For these actions, 5-HT2A receptors signaled through their canonical pathways of endoplasmic reticulum-mediated calcium release and 5-HT1B through cAMP depletion.

### 5-HT3 Receptors

The 5-HT3 receptors, the only ligand-gated, non-selective cation channel 5-HT receptors, are expressed in the cerebral cortex, hippocampus, amygdala, and the solitary tract nucleus (196). The 5-HT3 receptor is not coupled to second-messenger cascades which makes it different from the other members of the 5-HT receptor family. The expression of 5-HT3 receptors in neuroblasts during brain development has suggested that they may play a role in neuronal differentiation and development (197). However, there is debate regarding the role of 5-HT3 receptors in neurite outgrowth, as 5-HT3 receptors may enhance dendritic spine formation in thalamic cultures (194), but not neurite outgrowth (198). For example, 5-HT3 receptors form a complex with the light chain of microtubule-associated protein 1B (MAP1B) and the tubulin cytoskeleton in dendrites and growth cones of hippocampal neurons during developmental (199). However, knockout of 5-HT3 receptor did not alter dendritic spines at baseline or following long-term depression in adult mice (200).

### 5-HT4 Receptors

5-HT4 receptors are implicated in the regulation of multiple physiological processes and are highly expressed in various regions of the limbic and in several basal ganglia components of the rodent brain (201–203). In transfected cell lines and primary neurons, 5-HT4 receptors primarily induce the cAMP pathway via Gs proteins (204), but can also signal to ERK activation via SRC protein kinase (205). *In vitro* studies showed that 5-HT4 receptor activation induces decreases in neurite outgrowth (206). By contrast, studies in the hippocampus also showed that 5-HT4 receptor activation enhances learning-induced hippocampal dendritic spine formation *in vivo* (207). 5-HT4 receptor activation was shown to rapidly trigger dendritic spine formation in hippocampal neurons (208) via G13-RhoA signaling pathway (209). Pharmacological studies showed that agonist-induced 5-HT4 receptor activation inhibits basal synaptic transmission and theta-burst LTP via GABAergic activation (210), while enhancing low-frequency induced hippocampal LTD (211). In contrast, 5-HT4 antagonist induced thalamostriatal spike timing-dependent LTD expression (212), while blocking 5-HT-induced late LTP in the amygdala (213). Therefore, the 5-HT4 receptor has a role in modulating synaptic transmission via the regulation of long-term plasticity. In addition, 5-HT4 receptors mediate SSRI-induced “dematuration” of adult hippocampal granule neurons implicated in behavioral actions (214, 215). Furthermore, the antidepressant actions of SSRI in depression models requires 5-HT4 receptors (216, 217). Interestingly 5-HT4 receptors have been implicated in rapid induction of hippocampal neurogenesis and rapid antidepressant actions (218). Since the above studies have used systemic 5-HT4 ligands, global 5-HT4 knockout mice, or slice preparations, the relative roles of 5-HT4 induced actions on region-specific synaptic transmission, neuroplasticity, and neurogenesis in its behavioral and cognitive actions remains to be clarified using tissue-specific gene knockout or drug delivery approaches.

### 5-HT6 Receptors

The 5-HT6 receptors are expressed in diverse brain areas including the olfactory tubercle, cortex, dorsal and ventral striatum, hippocampus, amygdala as well as choroid plexus (219–221), and are implicated in schizophrenia, anxiety, and Alzheimer's disease (222). The 5-HT6 receptor activates ACs by coupling to Gs proteins (223), interacts with Fyn kinase to mediate ras-MEK-ERK1/2 signaling (224, 225), and with Jab1 to couple to the transcription factor c-Jun (226). *In vivo* studies in the developing cortex have implicated 5-HT6 receptors localized in dendritic cilia in dendritic outgrowth and neuronal differentiation, signaling via the Fyn pathway (227, 228). Actions of 5-HT6 signaling on neurite outgrowth involve constitutive activation of the receptor by cdk5, which can be blocked by 5-HT6 antagonist (229, 230). In addition, 5-HT6 signaling regulates migration of cortical pyramidal neurons and interneurons during development (231, 232). More recently, 5-HT6-/- mice have been shown to have altered *in vivo* dendritic and neuronal morphology, increased neuronal excitability, and increased anxiety and cognitive impairment phenotypes (233). In terms of neurotransmission, agonist-induced 5-HT6 receptor activation acutely increases expression of BDNF and Arc in cortical and hippocampal brain areas (234) and in the hippocampal CA1 area increases GABA release and decreases synaptic plasticity (235, 236). Using a 5-HT6 receptor antagonist increases the levels of glutamate, acetylcholine, and catecholamine in the frontal cortex and hippocampus and results in enhanced excitatory neurotransmission. 5-HT6 receptor antagonists inhibit the mTOR complex, which promotes neuronal survival and increases neurite outgrowth (237). This 5-HT6 modulation of the mTOR complex provides a promising target to treat anxiety, schizophrenia, and Alzheimer's disease (230). Although an increasing body of studies indicates that acute effects of both 5-HT6 receptor antagonists and agonists elicit improvement in depression and anxiety observed in the preclinical models (238), the underlying mechanisms are not clear. Given the importance of 5-HT6 receptors in cortical development, this receptor may also play a role in the recovery and regeneration of 5-HT projections lost in adulthood and associated with cognitive impairment (239) and depression (238).

### 5-HT7 Receptors

The 5-HT7 receptors are involved in the sleep-wake cycle, body temperature, depression-like behavior and the processes of learning and memory (240–242). In the brain, they are mainly expressed in the thalamus, hypothalamus, hippocampus, prefrontal cortex, amygdala, lateral habenula, raphe nuclei, and the suprachiasmatic nucleus (240, 243, 244). 5-HT7 receptors couple to Gαs (245) and Gα12 (206). Gα12/13 proteins signal to activate JNK, G protein signaling proteins (RGS) (246), non-receptor tyrosine kinases (nRTK) to signal to the Rho family of small GTPases that promote neurite extension and branching (247, 248). *In vitro* studies reported the involvement of 5-HT7 receptors in neurite outgrowth, spino- and synaptogenesis in young neurons, and increases in axon outgrowth via mTOR, Cdc4, to regulate actin filaments dynamics and metalloproteinase induced synaptic remodeling (249–252). Interestingly, in postnatal development, the 5-HT7 receptor is co-expressed with 5-HTT on PFC neurons and mediates PFC projections to the DRN implicated in development of anxiety and depression like phenotypes in mice treated postnatally with SSRI (253). The role of 5-HT7 receptors in modifying PFC projections during adulthood remains to be elucidated.

## Triggering 5-HT Axonal and Neurite Outgrowth

How is spontaneous 5-HT axonal outgrowth triggered? By analogy with activity-dependent neuroplasticity following stroke (254, 255), it is hypothesized that regrowth of 5-HT axons after an injury is driven by the activity of the affected 5-HT neurons. For example, following a stroke to the left mPFC, we have observed a 3–4 fold chronic activation (FosB^+^ cells) of dorsal raphe 5-HT and vGlut3-positive neurons that is maintained after fluoxetine treatment (256). Since full recovery of 5-HT innervation and behavior is only seen after chronic fluoxetine treatment (64), this suggests that fluoxetine-induced augmentation of 5-HT levels at target regions is critical for 5-HT innervation to mediate behavioral and cognitive recovery. This implicates 5-HT autoregulatory effects on its own axons in affected area, which may be mediated via 5-HT receptor signaling (as discussed above). The importance of 5-HT neuronal activation is suggested by deep brain stimulation of the mPFC in rats subjected to chronic social defeat. Increasing cortical drive to the raphe induced dendritic remodeling of 5-HT neurons to restore their activity, resulting in increased size and number of presynaptic 5-HT terminals in the hippocampus (66). The released 5-HT likely signals through a variety of 5-HT receptors on 5-HT projections, local glial cells and target neurons to ultimately restore behavior as discussed above.

## Detecting 5-HT Axonal and Neurite Outgrowth in Humans

Does the loss of 5-HT innervation occur in clinical depression, and can it be reversed by chronic SSRI treatment? The problem is how to visualize 5-HT innervation in depressed patients. One method is to use the 5-HTT as a biomarker for 5-HT innervation. In post-mortem brain, several regions show reduced 5-HTT staining including the ventral PFC, which was associated with depression and childhood maltreatment (257, 258). More specifically, visualization and quantification of 5-HTT-immunopositive processes have shown a reduction in the length of 5-HT axons in orbitofrontal cortex from depressed subjects (32). This region integrates multi-model sensory input to drive reward and affective behavior (259), and its activity is inversely correlated with the severity of depression (260). In living patients, this has been evaluated in positron emission tomography studies using ligands such as ^11^C-DASB. This is supported by the loss of DASB binding in cortex and striatum following MCAO in rats, with a gradual recovery over 3 weeks (128), similar to the time course that we observed in post-ischemic mice (64). Using ^11^C-DASB to label 5-HTT, a reduced 5-HTT ratio between dorsal raphe/ventral striatum was seen in unmedicated depressed compared to healthy controls, suggesting reduced 5-HT innervation to this reward processing center (261). By contrast, no difference in 5-HTT levels was seen in recovered depressed subjects compared to controls (262), whereas alterations are seen in several brain areas of severely depressed patients (263). Restorative effects of antidepressant treatment on 5-HTT levels have also been reported. In depressed subjects, altered 5-HTT ratio between median raphe to bilateral habenula, amygdala-hippocampus and subgenual cingulate cortex predicted treatment response (264). In bipolar depression, lower levels of 5-HTT and 5-HT1A predicted response and remission to 8-wk lithium treatment (265). Taken together, these studies suggest that alterations in 5-HTT levels, perhaps due to altered 5-HT innervation, are associated with depression and response to SSRIs. However, these changes could simply reflect changes in 5-HTT expression level, rather than 5-HT innervation. Functional connectivity studies using fMRI with the raphe as a seed may provide addition evidence of impaired 5-HT projections, as raphe connectivity strength mirrors 5-HTT levels in healthy controls (266). Acute tryptophan depletion decreased functional connectivity of the raphe to right pregenual anterior cingulate cortex in SSRI-resistant depressed subjects, but increased raphe-left thalamus connectivity in SSRI-responders, suggesting that increased 5-HT innervation correlates with SSRI response (267). Taken together, these studies indicate a deficiency in 5-HT innervation occurs in major depression and can be modified by chronic treatment in SSRI-responders. The importance in behavior of these neuroplasticity changes remains to be addressed but developing strategies to enhance 5-HT neuroplasticity may provide a more robust antidepressant response. Using models of SSRI-resistant depression such as the cF1ko mice (10), it will be possible to elucidate whether changes in 5-HT axons associated with depression and anxiety are unresponsive to fluoxetine and develop alternative or augmentation therapies to efficiently enhance the activity of 5-HT system and axonal plasticity to treat SSRI-resistant patients.

## Conclusion

Although not extensively studied, increasing evidence is indicating that deficiencies in 5-HT innervation associated with development, chronic stress or brain injury may lead to depression (10). Furthermore, the 5-HT system is capable of regenerating lost projections. Particularly after injury or chronic stress, 5-HT rewiring is induced during recovery (62), and can be enhanced by SSRI treatment or activation of 5-HT neurons (64, 66). While 5-HT rewiring correlates with recovery (66, 256), it remains to be directly addressed how important this mechanism is for recovery in rodent models. In humans, some research shows alterations in 5-HTT labeling in post-mortem OFC associated with major depression (32). Further studies are needed to determine what other brain regions might be affected, how early, and the effect of successful treatment on these projections.

Exactly how SSRIs might trigger reinnervation remains unclear. For example, 5-HT1A receptor-mediated induction of BDNF has been implicated in cortical synaptic plasticity, but whether BDNF mediates changes in innervation is unclear (188). However, abundant evidence indicates that several 5-HT receptors have actions to enhance synaptic plasticity and the formation of new synaptic connections. Direct activation of some of these receptors has been shown to mediate antidepressant actions in some tests and certain models of depression. However, it remains unclear how effective these compounds will be in human depression.

By coordinately targeting 5-HT activity, 5-HT release and 5-HT receptor-induced synaptic remodeling may provide a more effective strategy to treat depression, even in treatment-resistant depressed subjects.

## Author Contributions

FV-A conceived and wrote the first draft, corrected revised version, and approved the final version. PA conceived and revised the first draft and finalized the manuscript. Both authors contributed to the article and approved the submitted version.

## Funding

This research was supported by a University of Ottawa Brain and Mind Research Institute Fellowship to FV-A and operating grant funding from the Canadian Institutes of Health Research to PA (PJT168948), and support for open access fees from the University of Ottawa Library.

## Conflict of Interest

The authors declare that the research was conducted in the absence of any commercial or financial relationships that could be construed as a potential conflict of interest.

## Publisher's Note

All claims expressed in this article are solely those of the authors and do not necessarily represent those of their affiliated organizations, or those of the publisher, the editors and the reviewers. Any product that may be evaluated in this article, or claim that may be made by its manufacturer, is not guaranteed or endorsed by the publisher.
